# YOLO-RBSD: an efficient and accurate rice blast spore detector based on improved YOLOv8

**DOI:** 10.1186/s13007-026-01526-5

**Published:** 2026-04-05

**Authors:** Chunhong Li, Dong Huang, Huiru Zhou

**Affiliations:** 1 College of Mathematics and Informatics, South China Agricultural University, Guangzhou, 510642 China; 2 Lab of Artificial Intelligence, Guangzhou Open University, Guangzhou, 510091 China; 3 Key Laboratory of Smart Agricultural Technology in Tropical South China, Ministry of Agriculture and Rural Affairs, Guangzhou, 510642 China

**Keywords:** Rice blast, Crop disease monitoring, Deep learning, Triplet attention, Spore detection

## Abstract

Rice blast is an important fungal disease caused by *Magnaporthe oryzae*, and the air-borne disease can erupt in a short time, causing large-scale yield losses. Rapid and accurate detection of rice blast spores in microscopic images is crucial for monitoring spore density in the field, guiding farmers in timely pesticide application for effective prevention and control. However, there is a lack of an efficient and accurate method for detecting rice blast spores under complex field conditions currently. Traditional machine learning algorithms are better suited for detecting a single category under controlled conditions and rely on manual feature extraction, which limits their transferability. Therefore, this paper proposed a novel Rice Blast Spore Detector based on You Only Look Once algorithm - YOLO-RBSD, which can effectively detect rice blast spores and three categories of impurity particles in microscopic images against complex field backgrounds. Firstly, the detector introduced the triplet attention mechanism on the basis of YOLOv8s, enhancing its ability to capture cross-dimensional features. In addition, in order to further reduce model parameters and enhance model speed, the detector replaced partial CSPDarknet53 to 2-Stage FPN (C2f) modules with Depthwise Separable C2f (DSC2f) modules, realizing the optimal model structure design. Finally, YOLO-RBSD achieved a mean average precision (0.5) of 96.1% and a macro F1 score of 92.6%, processing 125 images per second, surpassing the mainstream models in both speed and accuracy. As an effective and lightweight tool, YOLO-RBSD provides a strong foundation for automated rice blast spore density monitoring in the field.

**Supplementary Information:**

The online version contains supplementary material available at 10.1186/s13007-026-01526-5.

## Introduction

Rice is one of the most important food crops worldwide, and according to the report of Food and Agriculture Organization of the United Nations [1], its production in 2023 ranked second only to corn. Rice blast, caused by *Magnaporthe oryzae*, is one of the most destructive diseases affecting rice cultivation, leading to significant yield losses and posing a serious threat to global food security [2]. The conidia of *M. oryzae* spread through airflow, germinating and colonizing healthy plants under suitable temperature and humidity conditions, a process that impacts growth and grain formation [3]. The density of airborne pathogen spores is closely linked to rice blast incidence, making spore monitoring crucial for predicting disease outbreaks and implementing timely control measures.

Traditionally, monitoring spore density involves field spore trapping experiments and subsequent biochemical experiments or manual counting under microscopes [4]. However, the biochemical methods demand high standards and expertise, and they are time-consuming, which makes them more appropriate for research than practical applications [5]. Meanwhile, impurities and spore deformation complicate microscopic analysis, and human error can lead to counting inaccuracies over time [6].

To address these challenges, spore detection using traditional machine learning has made progress, focusing on segmenting spores from microscopic images and designing features based on their morphology, texture, and color [7]. However, these methods struggle with closely spaced spores, and manually extracted features lack transferability, particularly as dataset complexity increases [8]. This limits the effectiveness of traditional algorithms in distinguishing different spore types.

In response, deep learning-based spore detection has gained attention due to its end-to-end training capabilities and effectiveness with large-scale datasets. Deep object detection algorithms fall into two categories [9]: two-stage algorithms like Faster R-CNN [10] and Cascade R-CNN [11], which extract candidate regions before classification and regression, and one-stage algorithms like SSD [12], RetinaNet [13], and YOLO [14], which directly output class and location information. One-stage algorithms are faster but may sacrifice some accuracy. However, advancements in YOLO have significantly improved its detection accuracy, leading to its widespread use in agriculture [15].

Recent advancements in the YOLO algorithm for fungal spore detection have showcased its effectiveness. Zhang et al. [16] enhanced YOLOv5s for detecting wheat scab fungus spores by adding the Efficient Channel Attention (ECA) mechanism and Adaptive Feature Fusion (ASFF) modules, which boosted accuracy but increased model parameters without improving detection speed. To address this, Yan et al. [17] replaced all C2f modules in YOLOv8s with FasterCSP modules for grapevine downy mildew sporangia detection, resulting in a smaller-size model. However, the Adaptive Cross Fusion Feature Pyramid Network (ACFFPN) module introduced in that study improved the mAP(0.5) by 2.3% from 88.6%, while reducing detection speed by over 30%. Similarly, Zhu et al. [18] improved YOLOv8 for detecting pathogenic fungi in cucumber diseases by introducing the global context attention module, the Content-Aware Reassembly of Features (CARAFE) upsampling module, and adding a small target detection layer, achieving a mAP(0.5) of 92.6%, but at the cost of increased computational load and reduced speed. To enhance both accuracy and speed, Li et al. [19] proposed an improved YOLOv5 algorithm for detecting cucumber gray mold spores, incorporating a multi-head self-attention module, weighted Bidirectional Feature Pyramid Network (BiFPN) and Ghost modules. This algorithm could process an image in 0.009 s with a detection accuracy of 0.983, but it only detects a single spore type, and its performance in natural scenes has yet to be validated.

**Fig. 1:**
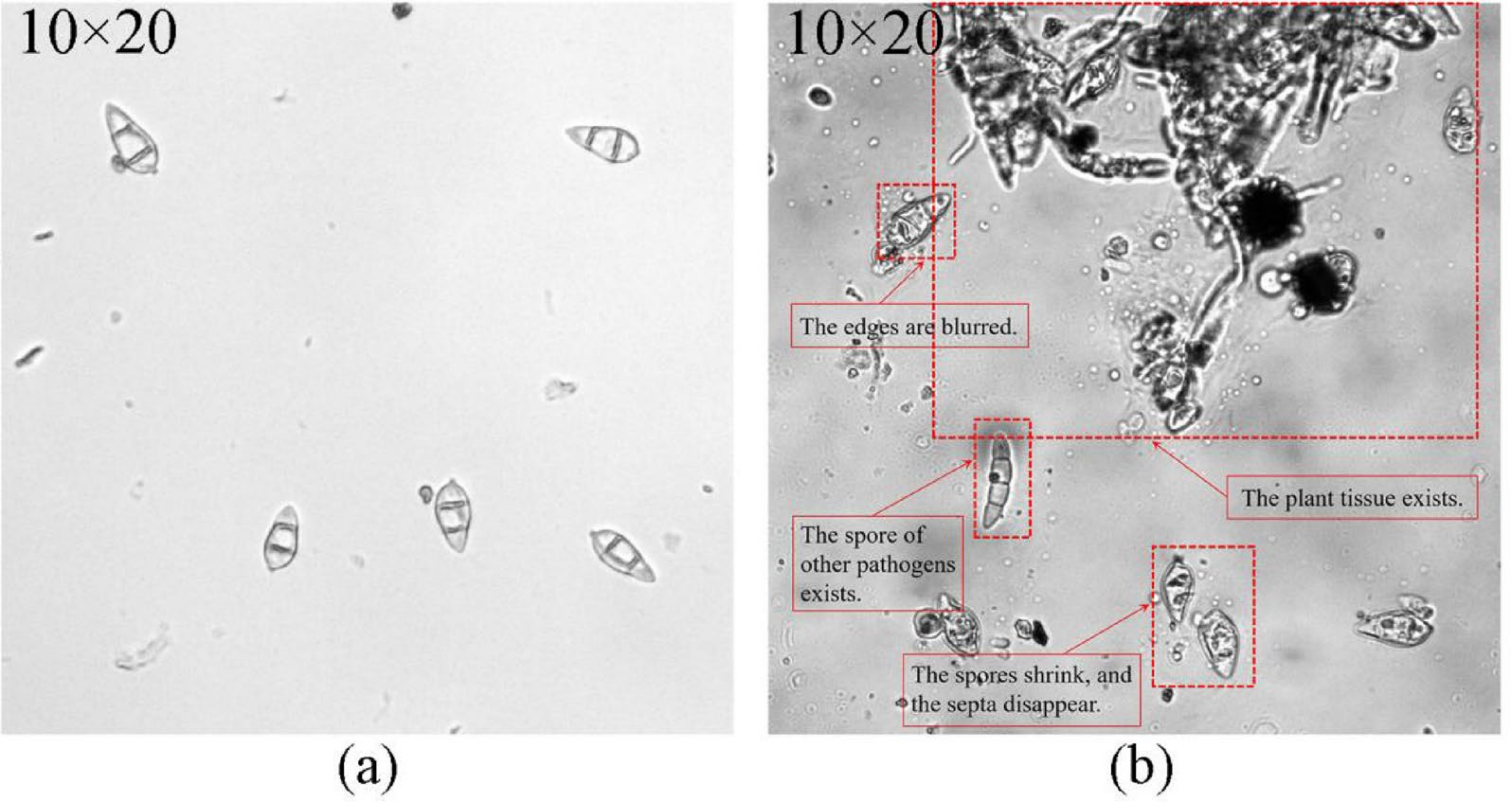
Comparison of (**a**) laboratory and (**b**) field-captured *Magnaporthe oryzae* spores at 200× magnification

Capturing rice blast fungus spores in the field is challenging due to equipment requirements, leading to greater difficulties in monitoring spore density. Research in this area is limited, with most studies relying on traditional machine learning methods focused on single categories in laboratory settings [20–22]. However, as shown in Fig. 1, spore slides collected from the field often contain various interfering factors, such as airborne spores from other diseases along with impurities like rice pollen, plant tissues, or small insects. Furthermore, the morphology of *M. oryzae* conidia from field samples differs from that of laboratory-cultured spores. For instance, they may exhibit only one septum or none at all (Fig. 1). Dehydration during the collection process can also lead to morphological shrinkage, which complicates the accurate identification of rice blast spores. Meanwhile, field-based spore collection is subject to natural variability. For instance, spore density fluctuates across different periods, and although spore overlap is generally low, adhesion between spores is often high. The types of interfering spores also vary with each collection. Collecting spores from different regions and time periods can therefore enhance the diversity of the dataset.

Since spores can disperse rapidly in the field, high detection efficiency is essential. Therefore, developing a real-time method for the accurate detection of rice blast spores has become an urgent priority. In previous studies on the detection of *M. oryzae* under lab conditions, experimental results have shown that single-stage YOLO series algorithms significantly outperform two-stage R-CNN series algorithms in speed, while their detection accuracy is only slightly lower than that of Cascade R-CNN. Therefore, it appears highly feasible to achieve superior accuracy by applying an improved YOLO algorithm [23].

Based on the aforementioned issues, this study aims to propose an improved version of the YOLOv8 algorithm and develop an accurate and efficient object detection model suitable for spore microscopic images. It is capable of efficiently processing the collected rice blast spore microscopic images in complex field environments and can identify and locate rice blast spores from several common types of fungal spores and rice pollen, thereby achieving the goal of spore counting. The main contributions of this paper are:

1. A dataset comprising 2000 in-field microscopic images of rice blast spores was constructed. It includes not only the conidia of rice blast fungus but also common interferents such as *Alternaria* spp. and *Fusarium* spp., as well as rice pollen. The dataset was meticulously organized, with standardized annotations, and was partitioned into training, validation, and test sets in a ratio of 8:1:1;
2. To enhance the feature extraction capability of the model for multi-class spores, a triplet attention mechanism was introduced into the junction between the P4 layer of backbone and the neck network. Consequently, the final model exhibited increases of 2.7% in AP, 2.0% in AR, and 4.2% in Macro-F1 score, respectively;
3. The original model architecture was optimized by replacing partial C2f modules of the backbone and all C2f modules of the neck network with lightweight DSC2f modules. This reduced the model parameters to 7.76 M and increased the inference speed to 125 frames per second;
4. The proposed YOLO-RBSD model demonstrated significant overall improvement over the baseline model YOLOv8s, achieving a leading advantage in comparison with other nine state-of-the-art object detection algorithms.

## Materials and methods

### Dataset construction

#### Field spore trapping experiment

**Fig. 2:**
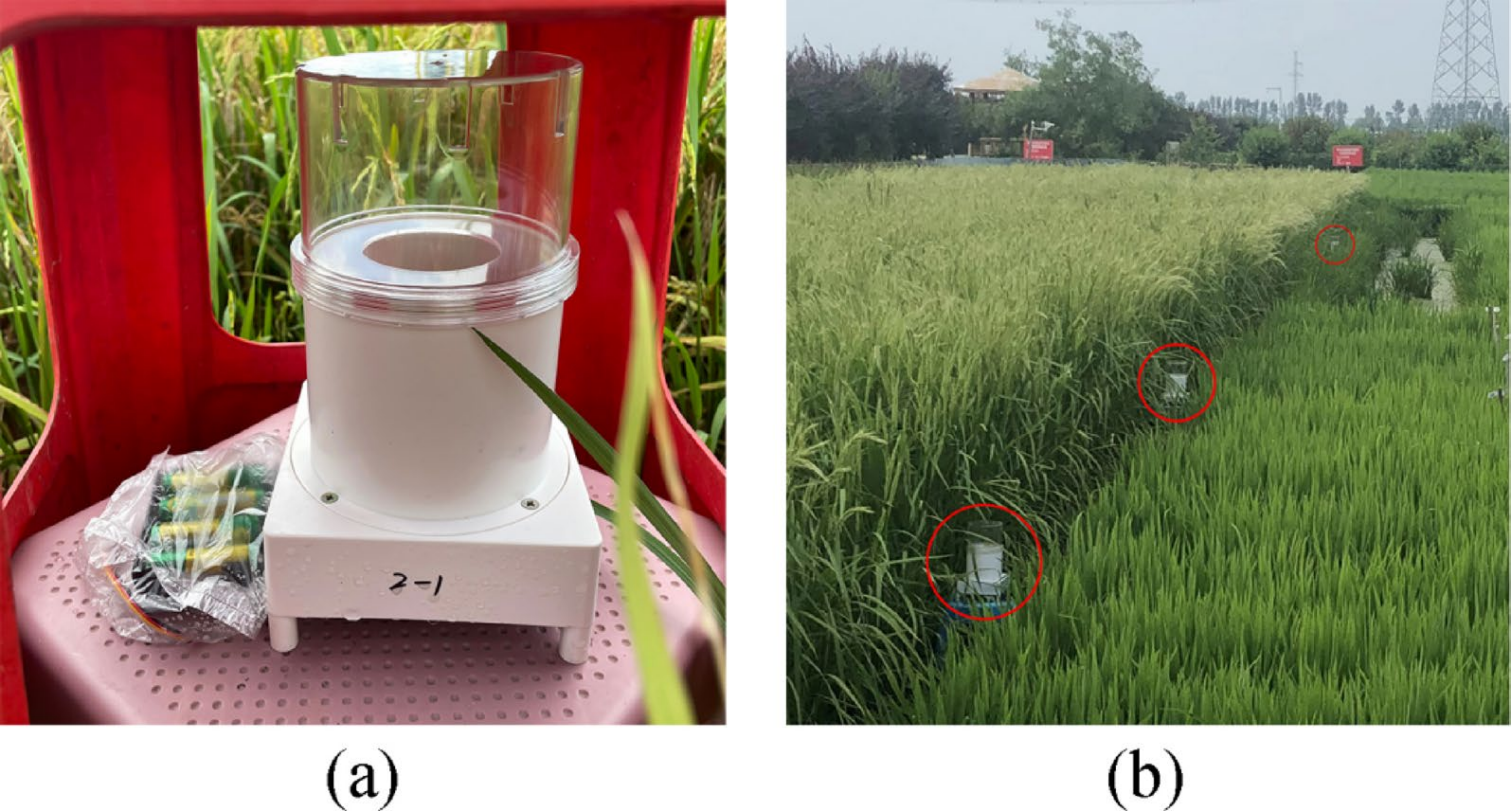
The main equipment used for field spore trapping experiments. **a** The front view of the portable spore trap, **b** The portable spore traps marked by red circles were placed in the field

To collect more representative field samples, this study selected three cities located in northern, central-southern, and southern China: Panjin City (Liaoning Province), Shangrao City (Jiangxi Province), and Yangjiang City (Guangdong Province). The collection points in the three cities are breeding bases for rice cultivation, where some disease-susceptible varieties are planted. Continuous spore trapping experiments were conducted for one week at the three experimental bases during the peak infection stages of leaf blast and panicle blast in 2020, 2021, and 2023. At each experimental base, two field plots were selected, with three to five spore traps placed in each field plot. The spore trap was provided by the Lab of Plant Disease Epidemiology at China Agricultural University, and its Chinese invention patent number is ZL201410009269.6 (Fig. 2a).

The collection mechanism involved airborne spores entering the traps via natural airflow and subsequently adhering to petroleum jelly-coated glass slides. Traps were strategically placed at the highly infected areas of field plots and operated from 4:00 PM to 9:00 AM the following day to optimize spore capture during peak dispersal periods (Fig. 2b).

#### Image acquisition and preprocessing

Image acquisition was conducted using integrated microscopic imaging equipment (Camera: Zeiss AxioCam ERc 5s Microscope camera, Manufacturer: Carl Zeiss AG, Oberkochen, Germany; Camera: MOTICAM ProS5 Plus, Manufacturer: Motic China Group Co Ltd, Xiamen, China). Initially, spore samples mounted on glass slides were examined under microscopes at 10 × 20 magnification to identify suitable fields of view for image capture. Subsequently, high-resolution images were captured in JPG format using the integrated imaging system.

Generally, the identification of fungal spores under a microscope relies primarily on morphological characteristics such as shape, septation, and size. For the four categories selected in this study, these morphological features are sufficient to meet the detection requirements according to the experience of human experts. Although some spores may exhibit color, this trait is often inconsistent, and many spores appear transparent or colorless. Moreover, variations in color temperature adjustments across different imaging devices can introduce interference due to inconsistent color rendering. Additionally, many imaging devices are only capable of producing grayscale images. Therefore, to ensure consistency across the dataset, all images were converted to grayscale.

Following curation, a final dataset comprising 2000 grayscale microscopic images of field spores was established. For systematic organization, the images were randomly shuffled and assigned sequential identifiers following the nomenclature “SP” + numerical index (e.g., SP(0) for the first image). This standardized naming convention facilitates efficient data management and retrieval during subsequent analyses.

#### Image annotation and dataset construction

**Fig. 3:**
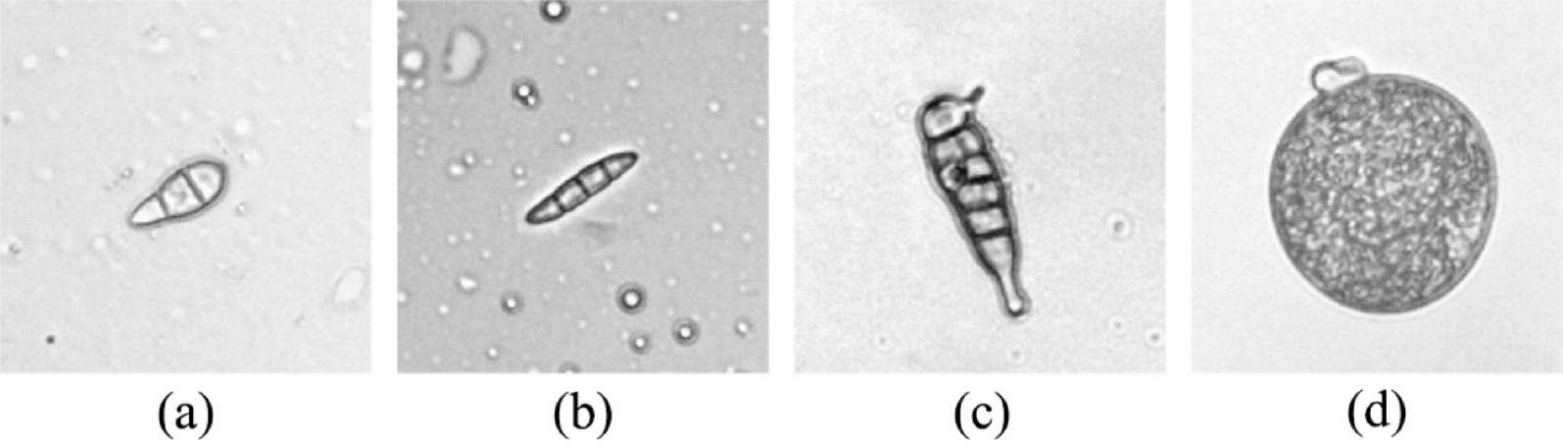
Examples of four categories in the microscopic image dataset. **a ***Magnaporthe oryzae*, **b ***Fusarium* spp., **c ***Alternaria* spp., **d** Rice pollon

Image annotation was performed using the open source data labeling tool - LabelImg, with all annotations saved in XML format. In addition to *Magnaporthe oryzae* (mag), common impurities including *Alternaria* spp. (alt), *Fusarium* spp. (fus), and rice pollen (pol) were unavoidably collected due to regional and seasonal variations (Fig. 3). To improve model classification performance, three impurity categories were also systematically annotated. The annotation process adhered to the following criteria: (1) Objects with ≥ 50% visible area were annotated; (2) Overlapping or adherent objects were labeled as distinct instances; (3) Morphologically indistinguishable objects were excluded from annotation.

The dataset was constructed in accordance with the YOLO dataset format. Initially, the annotation files were converted into TXT format. Subsequently, the 2000 images with their paired annotations were randomly partitioned into training (80%), validation (10%), and test (10%) sets. Detailed dataset specifications are provided in Table 1.

**Table 1:** The details of microscopic image dataset used in this study

Train set	Image quantity	Instances (alt)^a^	Instances (fus)^b^	Instances (mag)^c^	Instances (pol)^d^
	1600	541	1379	1559	1322
Validation set	200	51	145	182	180
Test set	200	57	189	202	172
Total	2000	649	1713	1943	1674

^a^*Alternaria* spp.

^b^*Fusarium* spp.

^c^*Magnaporthe oryzae*.

^d^Rice pollon

**Fig. 4:**
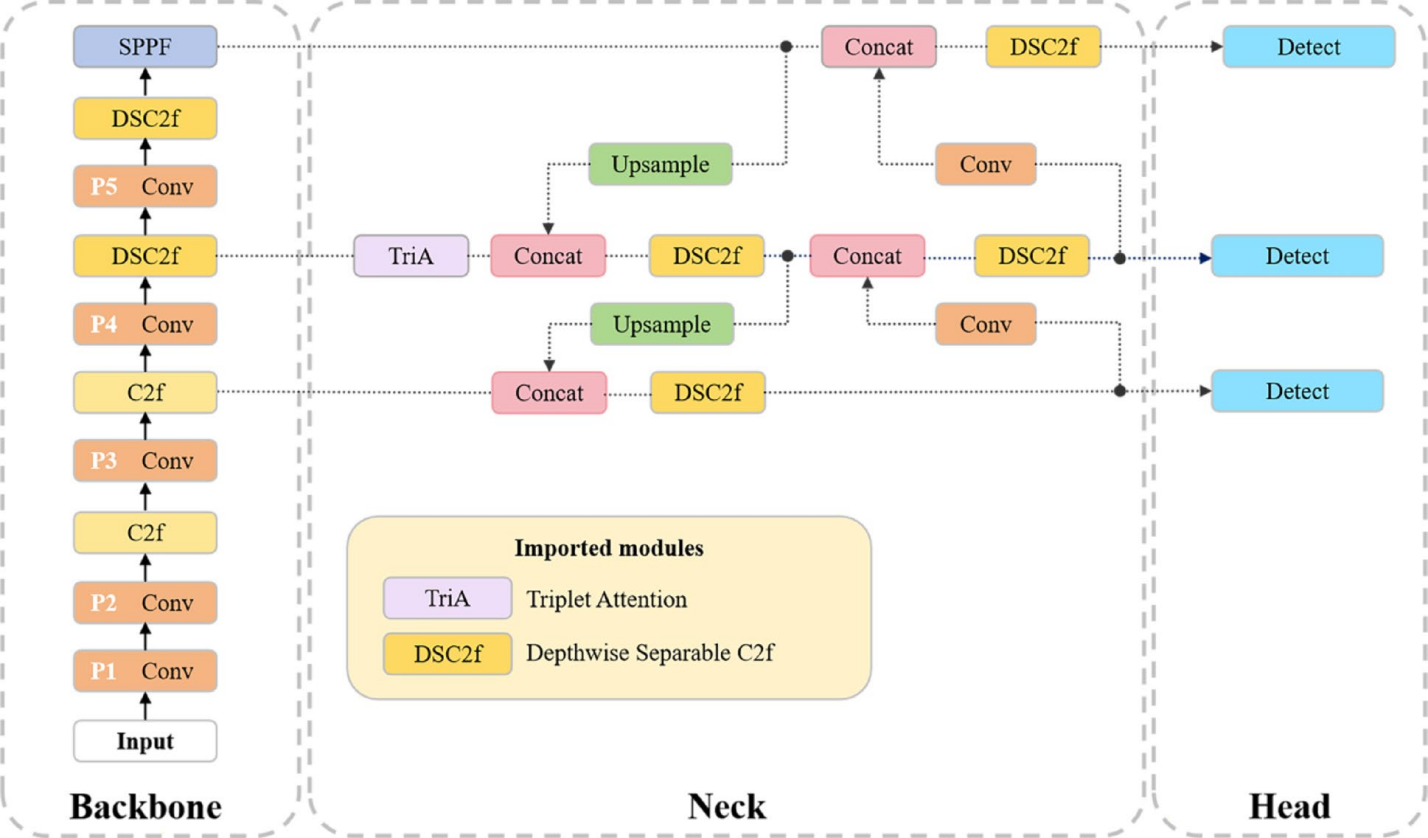
The architecture of the proposed YOLO-RBSD network. It consists of three primary components: (1) the backbone, where the original C2f modules in its deeper layers (e.g., P4, P5) are replaced with our lightweight DSC2f modules, (2) the neck, which is entirely constructed with DSC2f modules and incorporates the triplet attention mechanism for spatial-channel feature refinement, and (3) the detection head for classification and bounding box regression

### YOLO-RBSD network

In this study, a novel detection framework YOLO-RBSD was developed for accurate and efficient identification of rice blast spores in microscopic images. Building upon YOLOv8 [24], the framework imported two key optimizations: Firstly, a triplet attention-guided [25] feature refinement mechanism was introduced to amplify discriminative spatial-channel interactions. Subsequently, DSC2f modules that leverage depthwise separable convolutions [26] were incorporated to eliminate computational redundancy and enhance the model inference speed. The overall architecture of YOLO-RBSD is illustrated in Fig. 4.

#### Motivations for baseline selection and brief introduction of YOLOv8

The original YOLO team developed three core versions, including YOLOv5 [27], YOLOv8 and YOLOv11 [28]. The iterative updates of these versions typically introducing the most state-of-the-art and highly effective modules, leading to breakthrough advancements. By analyzing structural modifications, it can be found that compared to YOLOv5, YOLOv8 mainly replaces the C3 structure with the C2f structure, and modifies the head section to a decoupled head with an anchor-free design, resulting in significant performance improvements. In contrast, the overall framework of YOLOv11 remains largely unchanged from YOLOv8. It primarily enhances feature extraction via the Convolutional block with Parallel Spatial Attention (C2PSA) module and reduces model parameters through the lightweight Cross Stage Partial with kernel size 2 (C3K2) module. This is an improvement strategy similar to that of YOLO-RBSD. Thus, optimizing YOLOv11 offers limited value, while YOLOv8 presents substantial potential for further performance gains.

YOLOv8 utilizes CSPDarknet53 as its backbone network and PANet [29] as the neck network, followed by anchor-free decoupled heads for object detection. The hierarchical design of the backbone generates feature maps at strides of 8, 16, and 32, denoted as P3, P4, and P5, respectively. These multi-scale features are then seamlessly integrated by the PANet through bidirectional top-down and bottom-up pathways. This fusion mechanism combines high-resolution shallow features, rich in spatial details, with low-resolution deep features, abundant in semantic context, ensuring superior performance in detecting objects across diverse scales. Furthermore, the decoupled head architecture enhances detection precision by segregating classification and regression tasks into distinct branches, each optimized with specialized loss functions Binary Cross-Entropy (BCE) for classification and Complete Intersection over Union (CIoU) for localization. This comprehensive design not only improves feature representation but also ensures high detection accuracy and efficiency, making YOLOv8 a powerful solution for real-time object detection tasks.

There are several versions of YOLOv8, including five preconfigured variants: nano, small, medium, large, and extra-large. Each variant features a network structure with varying depths and widths, designed to accommodate different hardware constraints.

#### Triplet attention for dimension-aware feature enhancement

**Fig. 5:**
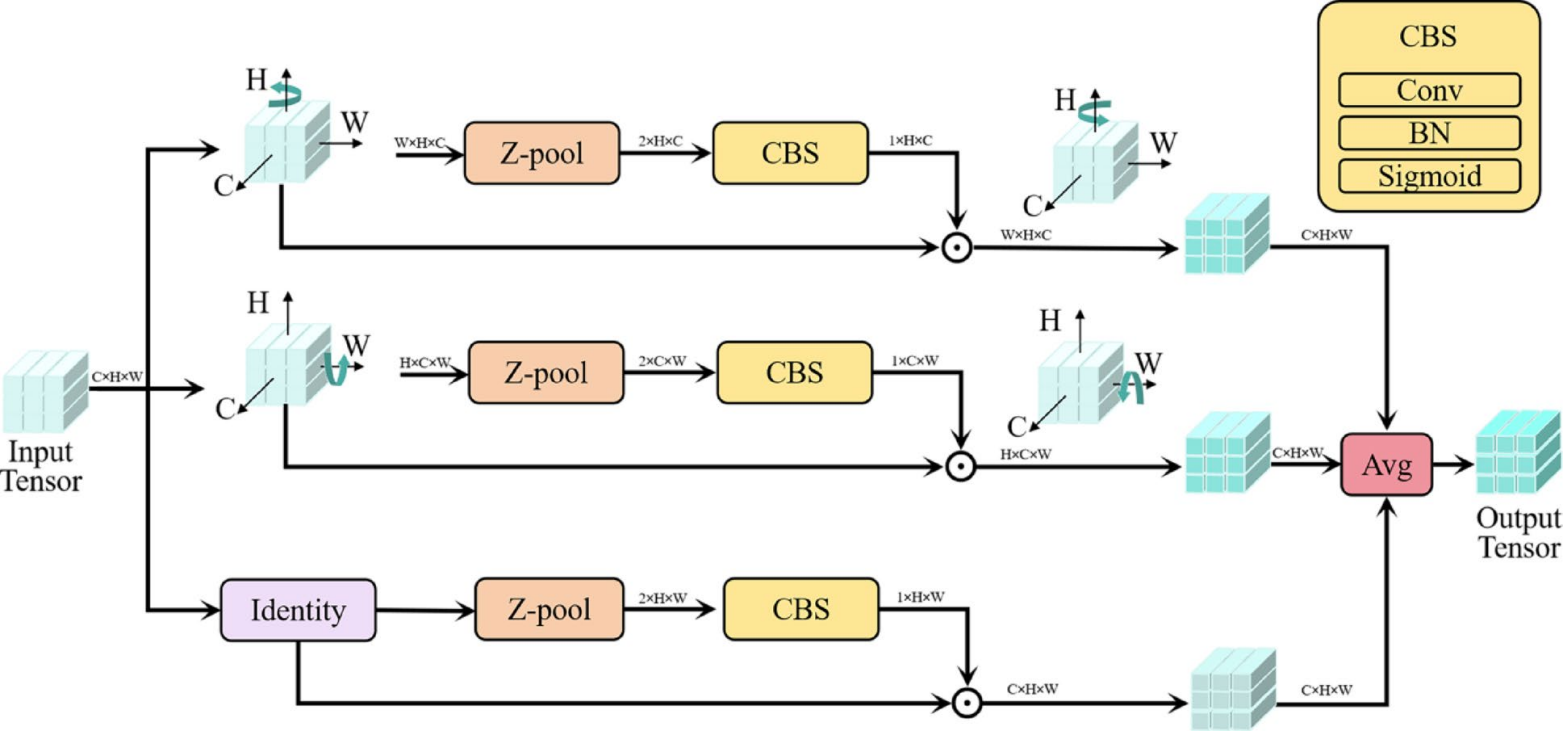
The structure of triplet attention module. The top branch establishes attention weights between channel C and spatial dimension W through rotational transformation, while the middle branch similarly connects channel C with spatial dimension H. The bottom branch directly models the spatial dependencies between dimensions H and W. These three complementary attention pathways are ultimately combined via weighted averaging to produce the refined feature representation

As shown in Fig. 5, the triplet attention module processes the input feature map $F \in {R^{C \times H \times W}}$ through three parallel branches, each is specifically designed to capture the dependencies between the (C, H), (C, W), and (H, W) dimensions, thereby enabling a comprehensive analysis of the input tensor. Here, R represents the set of real numbers, while C, H, and W denote the channel, height, and width of the feature map, respectively. In the first branch, we establish interactions between the height and channel dimensions. To achieve this, we first perform a 90° anti-clockwise rotation of the input tensor $F$ along the H axis, resulting in a transformer tensor ${\hat {F}_1}$ with the shape (W×H×C). Then the ${\hat {F}_1}$ is passed through the Z-pool layer to yield $\hat {F}_{1}^{*}$, which is of shape (2×H×C). The Z-pool can be represented as follows:

1 $$ Z - pool(\hat{F}_{1} ){\text{ }} = {\text{ }}[Maxpool_{{0d}} (\hat{F}_{1} ),{\text{ }}AvgPool_{{0d}} (\hat{F}_{1} )] $$

Where 0d represents the 0th dimension across which the max and average pooling operations occur. The processed tensor $\hat {F}_{1}^{*}$ is sequentially subjected to a standard convolution layer of kernel size 7 × 7 (${\mathrm{Con}}{{\mathrm{v}}_{{\mathrm{7}} \times {\mathrm{7}}}}$), followed by batch normalization (BN) to obtain an intermediate tensor (1×H×C). Subsequently, a sigmoid activation function(σ) is applied to this intermediate tensor to obtain the channel-height attention (${A_{{\mathrm{ch}}}}$) weights as follows:

2 $${A_{{\mathrm{ch}}}}=\sigma (BN(Con{v_{7 \times 7}}(\hat {F}_{1}^{*})))$$

Finally, the first branch output (${F_{{\mathrm{out}}1}}$) is represented as follows:

3 $${F_{out1}}=RC_{H}^{{90}}({\hat {F}_1} \odot {A_{{\mathrm{ch}}}})$$

where ☉ denotes broadcast element wise multiplication and $RC_{H}^{{90}}$ represents rotating 90° clockwise along the H axis, respectively.

Similarly, the input F is rotated 90° anti-clockwise along the W axis in the second branch, resulting in ${\hat {F}_2}$ with a dimension configuration of (H×C×W). Furthermore, through the Z-pool layer, ${\hat {F}_2}$ is transformed into $\hat {F}_{2}^{*}$, whose shape becomes (2×C×W). Likewise, $\hat {F}_{2}^{*}$ is fed into a convolution layer of kernel size 7 × 7, followed by a batch normalization (BN) step to gain an intermediate tensor (1×C×W). Consequently, the channel-width attention (${A_{cw}}$) weights are acquired by passing the intermediate tensor through a sigmoid activation process (σ), as follows:

4 $${A_{{\mathrm{cw}}}}=\sigma (BN({\mathrm{C}}on{v_{7 \times 7}}(\hat {F}_{2}^{*})))$$

The weights are then employed on ${\hat {F}_2}$, and the output is subsequently rotated 90° around the W axis ($RC_{W}^{{90}}$) to yield the final output of the second branch (${F_{{\mathrm{out}}2}}$), as follows:

5 $${F_{out2}}=RC_{W}^{{90}}({\hat {F}_2} \odot {\mathrm{A}_{\mathrm{c}\mathrm{w}}})$$

The final branch, different from the previously mentioned two branches, diverges by bypassing the rotation of the input $F$. It proceeds directly through a Z-pool layer to produce a tensor $F_{3}^{*}$ with a shape of (2×H×W). Following this, the tensor $F_{3}^{*}$ is subjected to a convolutional layer with a kernel size of 7 × 7, followed by a Batch Normalization (BN) layer and a sigmoid activation layer, thus deriving the height-width attention weights (${A_{{\mathrm{h}}w}}$) as follow:

6 $${A_{hw}}=\sigma {\mathrm{(}}BN{\mathrm{(}}Con{v_{7 \times 7}}{\mathrm{(}}F_{3}^{*}{\mathrm{)))}}$$

Consequently, the output of the third branch can be formally represented as follows:

7 $${F_{out3}}=F \odot {A_{hw}}$$

Ultimately, the refined attention-applied tensor $F^{\prime}$ derived from triplet attention mechanism can be represent by the following equation:

8 $$F^{\prime}=\frac{1}{3}({F_{out1}}+{F_{out2}}+{F_{out3}})$$

Consequently, we obtain the ${P^{\prime}4}$ layer and the multi-scale features {$P3$, ${P^{\prime}4}$, $P5$ } serves as the input layer for the neck network PANet.

#### DSC2f: Lightweight bottleneck design with depthwise separable convolutions

**Fig. 6:**
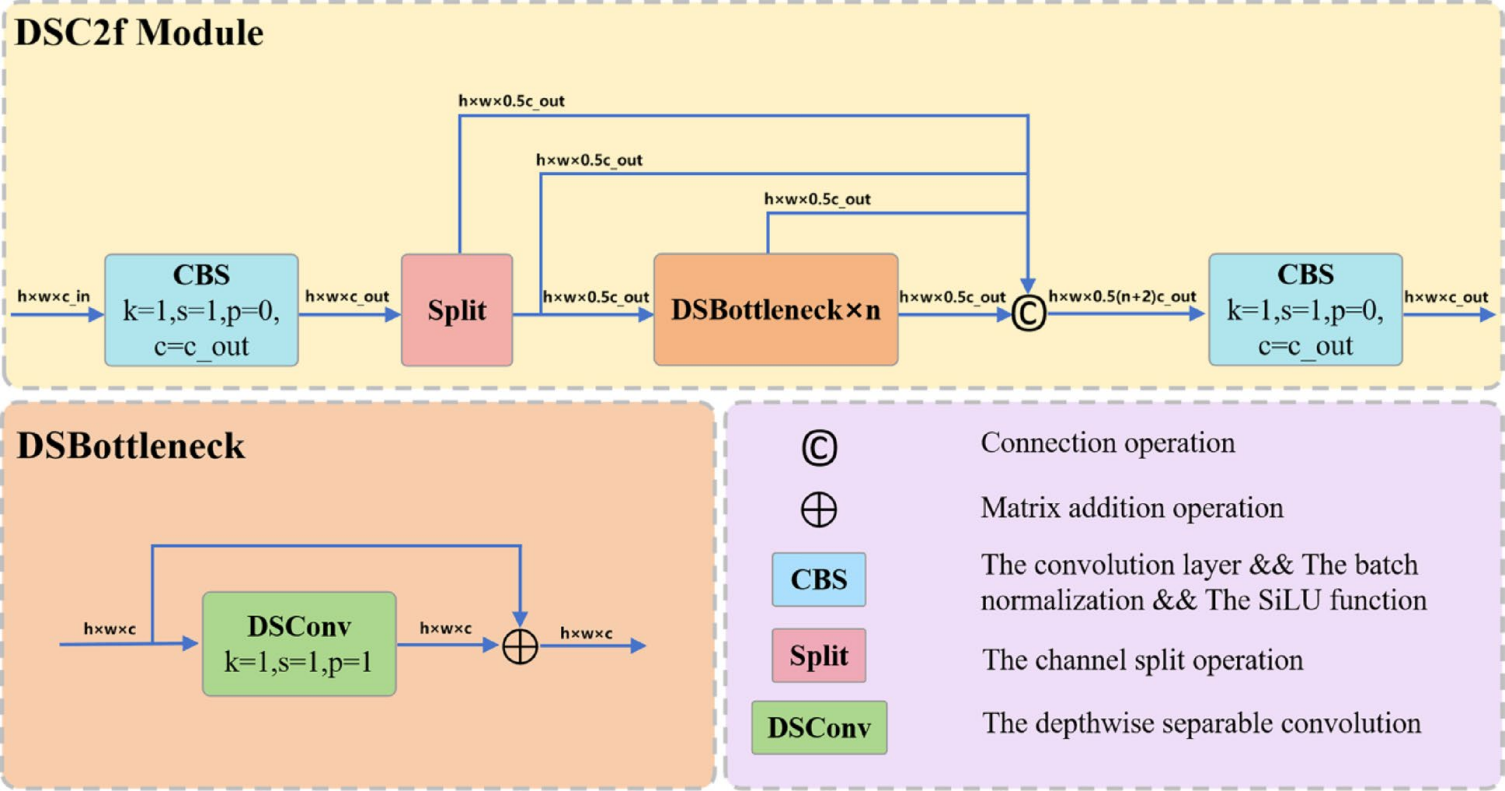
The structure of the lightweight DSC2f module. The input feature is first projected by a 1 × 1 CBS block and then split along the channel dimension into two paths. One path is preserved as a skip connection, while the other not only serves as a skip connection but also undergoes feature extraction through n sequential DSBottleneck residual units. The *n* + 2 branches are then concatenated and integrated by a final 1 × 1 CBS block to produce the output

In this section, we propose the Depthwise Separable CSPDarknet53 to 2-Stage FPN (DSC2f) module, a lightweight bottleneck design that leverages depthwise separable convolution to replace the two 3 × 3 convolutions in the DarkNet bottleneck of original C2f module. The structure of DSC2f module is shown in Fig. 6.

As shown in Fig. 6, given an input feature map ${F_{{\mathrm{in}}}} \in {R^{C \times H \times W}}$, the DSC2f module processes it through the following structured pathway to obtain a refined output feature. Firstly, the input feature is processed using a 1 × 1 convolutional block architecture, which includes a 1 × 1 convolution layer, a batch normalization layer, and a SiLU activation function (CBS), the equation can be represented as follows:

9 $${F_0}=S{\mathrm{i}}LU\left( {BN\left( {C{\mathrm{on}}{{\mathrm{v}}_{1 \times 1}}\left( {{F_{{\mathrm{in}}}}} \right)} \right)} \right)$$

where ${F_0} \in {R^{{C_{{\mathrm{out}}}} \times H \times W}}$. Subsequently, the extracted feature ${\it \:\mathrm{F}}_{\mathrm{0}}$ is directed to a split node, where it is uniformly split into two parts along the channel dimension, as follows:

10 $$ F_{{skip}} ,F_{{proc}} = Split{\text{ }}(F_{0} ) $$

Consequently, the processing branch ${F_{proc}}$ is further divided into two streams. One retains the input feature of the residual structure, and the other extracts features through n DSBottleneck units. Each DSBottleneck unit employs a depthwise separable convolution and a residual addition, maintaining the channel dimension throughout. The output of the i-th DSBottleneck unit (i = 1, …, n) can be represented by the following equation:

11 $$ F_{i}^{{ds}} = F_{{i - 1}}^{{ds}} \oplus DSConv_{{1 \times 1}} \left( {F_{{i - 1}}^{{ds}} } \right) $$

where $F_{0}^{{{\mathrm{ds}}}}={F_{{\mathrm{proc}}}}$, and $\oplus$ denotes matrix addition operation. At the end of the bottleneck, the feature maps of all branches are concatenated to obtain , as follows:

12 $$F^{\prime}={F_{{\mathrm{skip}}}}+{F_{proc}}+\sum _{{i=1}}^{n}F_{i}^{{ds}}$$

Where $F^{\prime} \in {R^{(0.5(\mathrm{n}+2){\mathrm{C}_{\mathrm{o}\mathrm{u}\mathrm{t}}} \times \mathrm{H} \times \mathrm{W})}}$. Finally, a 1 × 1 CBS block is applied to integrate the concatenated features and produce the output feature map with the desired channel count ${C_{out}}$:

13 $${F_{{\mathrm{out}}}}=S{\mathrm{i}}LU\left( {BN\left( {C{\mathrm{on}}{{\mathrm{v}}_{1 \times 1}}\left( {F^{\prime}} \right)} \right)} \right)$$

Through this design, the DSC2f module effectively reduces computational complexity and parameter count by utilizing depthwise separable convolutions, while preserving rich multi-scale feature representations via its split-transform-merge structure.

### Experimental details

**Fig. 7:**
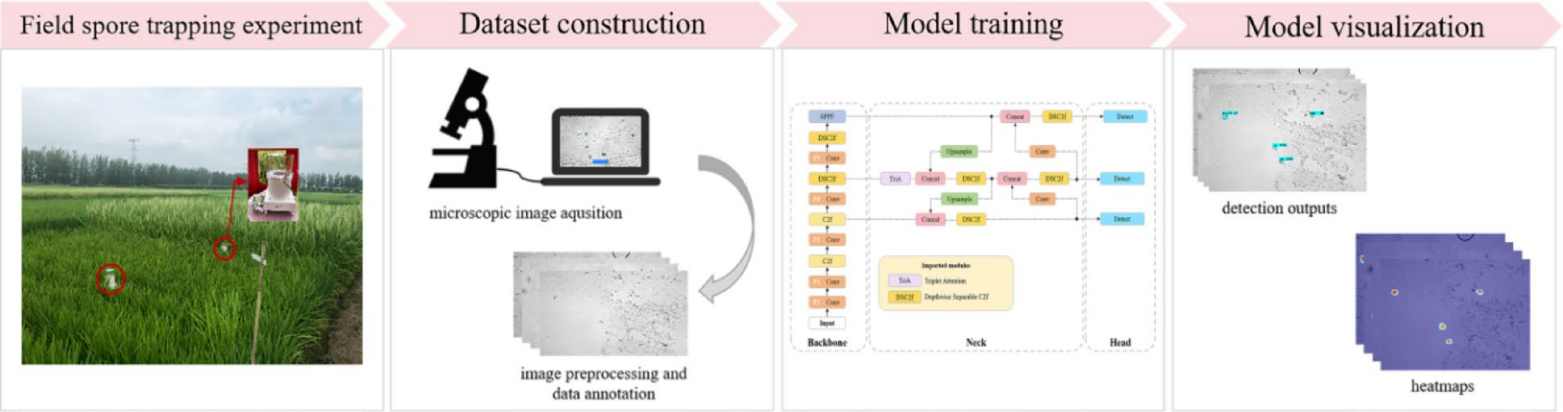
Flowchart of the overall experiment in this study

In this study, to experimentally compare the proposed model against other models, each model was trained for 100 epochs with a batch size of 32. It is important to note that the transfer learning approach was employed to train the model, with the pre-trained weights loaded from the COCO dataset. This method could avoid random initialization of model parameters, accelerating convergence and conserving computational resources (Fig. S1). After training, weights were saved every ten epochs, and the weight with the smallest loss was selected as the target weight. Additionally, the optimizer used was Stochastic Gradient Descent (SGD), with an initial learning rate of 1e-2 and a weight decay of 5e-4. The learning rate decay followed a cosine annealing strategy. The input image resolution was maintained at 640 × 640 throughout the experiments. For a fair comparison, all models utilized Non-Maximum Suppression (NMS) with a threshold of 0.5 to eliminate duplicate detections. The experiments were conducted on an NVIDIA GeForce RTX 2080Ti GPU by using PyTorch version 1.11.0. The overall workflow of the experiment is depicted in Fig. 7, comprising four main phases: the field spore collection, dataset construction, model training, and model visualization.

### Evaluation indicators

Indicators for evaluating the accuracy of object detection algorithms typically include precision (Eq. 14), recall (Eq. 15), F1 score (Eq. 16). Precision refers to the proportion of true positive samples among all samples predicted as positive. Recall refers to the proportion of correctly predicted positive samples relative to all actual positive samples. The F1 score represents the harmonic mean of precision and recall. A higher F1 score, approaching 1, indicates superior overall model performance.

14 $$Precision=\frac{{TP}}{{TP+FP}}$$

15 $$\operatorname{Re} call=\frac{{TP}}{{TP+FN}}$$

16 $$ F1 = 2\frac{{Precision \times Recall}}{{Precision + Recall}} $$

*TP*: True positive indicates the number of positive samples accurately predicted. *FP*: False positive indicates the number of negative samples incorrectly predicted as positive. *TN*: True negative indicates the number of negative samples accurately predicted. *FN*: False negative indicates the number of positive samples incorrectly predicted as negative.

Meanwhile, the Intersection over Union (IoU) threshold (Eq. 17) is a key metric for assessing the accuracy of model localization, and its configuration will also affect the detection results.

17 $$I{\mathrm{o}}U({A_P},{A_G})=\frac{{{A_P} \cap {A_G}}}{{{A_P} \cup {A_G}}}$$

*A*_*P*_ means the area of the prediction box, *A*_*G*_ means the area of the ground truth.

Since this study involves multi-class object detection, the Microsoft Common Objects in Context (MS COCO) evaluation system (http://cocodataset.org) was used to evaluate the overall performance of the model. Specifically, Average Precision (AP) and AP(50) were selected as indicators for precision, while Average Recall (AR) with a maximum of 100 objects was chosen as an indicator for recall. AP is computed as the mean of average precision values at IoU thresholds ranging from 0.5 to 0.95 with a step size of 0.05, also commonly expressed as mAP(0.5:0.95). And many studies also refer to AP(50) as mean Average Precision (IoU ≥ 0.5) (mAP(0.5)). The comprehensive evaluation indicator, the macro F1 score, is introduced and calculated using Eq. 18.

18 $$macro\_F1\_score=\frac{{\sum\limits_{{i=1}}^{C} {F{1_i}} }}{C}$$

*C* represents the number of categories, and *F*1_*i*_ represents F1 score of the *i*_*th*_ category.

To evaluate the real-time performance of the model in detection tasks and its impact on spore monitoring efficiency, this study adopts inference speed as the evaluation metric, quantified by frames per second (FPS). All speed tests were conducted on the test set under identical hardware conditions. The specific parameter settings are as follows: the input image resolution was uniformly resized to 640 × 640, the batch size was set to 1, and inference was performed on a single GPU using FP32 precision within the PyTorch framework. To ensure the authenticity of the evaluation, the inference pipeline comprises the model forward pass and the non-maximum suppression (NMS) post-processing stage, but excludes image preprocessing. During testing, no warm-up operations were performed, in order to reflect the actual processing speed of the model under cold-start conditions.

In addition to FPS, we also selected two additional indicators, model size and computational load (GFLOPs), to further assess our lightweight model. A model that can meet the requirements for real-time applications should have fewer parameters, lower inference time, and faster image processing speed.

## Results and analysis

### Baseline model variant selection

A preliminary experiment was performed to evaluate multiple variants of the baseline model YOLOv8 using the self-built dataset (Table 2). The results demonstrate a positive correlation between model complexity and detection performance, as reflected in key indicators such as mAP(0.5), AP, AR, and macro F1 score. However, this improvement in accuracy was accompanied by a significant reduction in detection speed. For instance, YOLOv8x, the largest variant, exhibits a model size approximately 20 times greater than that of YOLOv8n (the smallest version), yet achieves less than one-third of its FPS. Importantly, a larger model scale does not always result in better detection performance. Despite its smaller size, YOLO8l outperformed YOLOv8x in this test, suggesting that YOLOv8x was designed for larger-scale datasets.

**Table 2:** Comparison of different variants of YOLOv8

Models	mAP(0.5)	AP	AR	Macro F1	Model size (M)	GFLOPs (G)	FPS (f/s)
YOLOv8n	0.915	0.648	0.727	0.851	3.16	8.86	125
YOLOv8s^※^	0.945	0.694	0.754	0.884	11.17	28.82	120
YOLOv8m	0.937	0.708	0.761	0.884	25.90	79.32	77
YOLOv8l	0.943	0.711	0.767	0.912	43.69	165.74	51
YOLOv8x	0.938	0.707	0.761	0.898	68.23	258.55	36

※: It was selected as a baseline model for further improvements

Given the objective of developing a rice blast spore detection model that balances accuracy and real-time performance, YOLOv8s was selected as the baseline model for further optimization. Compared to YOLOv8n, YOLOv8s offers similar model size and inference speed, but with significantly enhanced detection performance. Furthermore, it surpasses the three larger variants (YOLOv8m, YOLOv8l, and YOLOv8x) in certain indicators, achieving the highest mAP(0.5) value (0.945) among all tested versions (Table 2).

### Validation experiments of triplet attention module

**Fig. 8:**
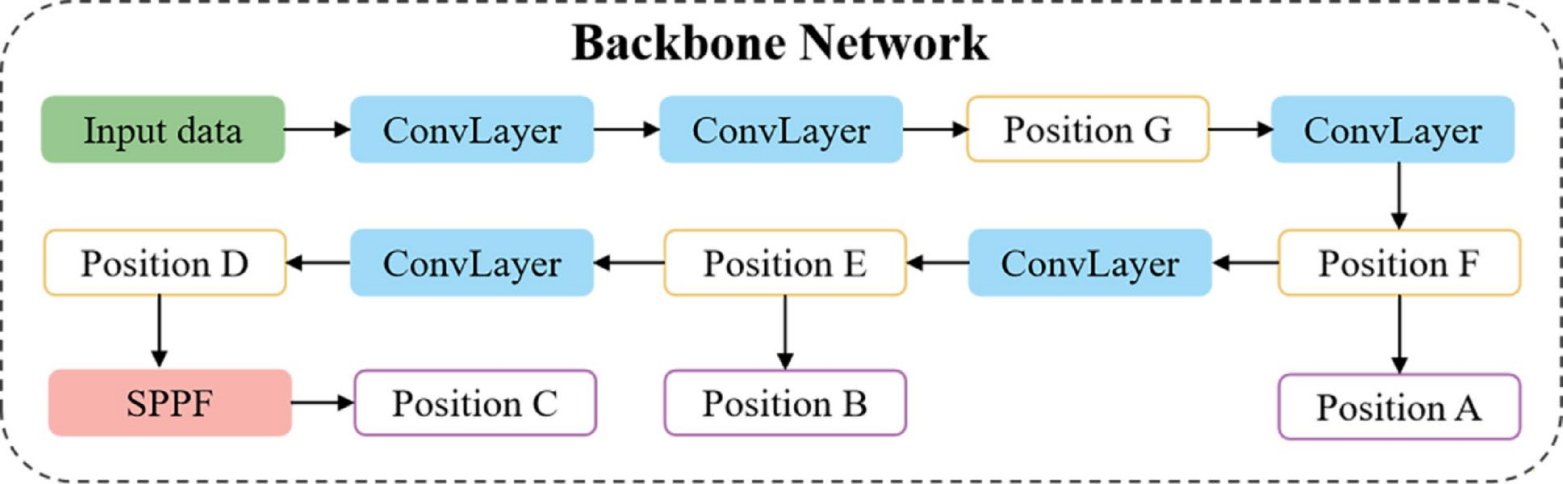
The potential positions in YOLOv8s backnone network for introducing two proposed modules. Positions A, B and C are the possible positions to insert the triplet attention modules; Positions D, E, F and G are the C2f modules of the backbone network that could be optimized to DSC2f modules. Besides, all C2f modules in the neck network will be optimized to DSC2f

Adding an attention mechanism to the backbone network may lead to the loss of original features. Therefore, introducing the triplet attention mechanism at the connection between the backbone network and the neck network was considered. The backbone network of YOLOv8s connects with the neck network at three positions: A, B and C, as illustrated in Fig. 8. The dimensions of the output feature maps vary across these connection positions, and the impact of the triplet attention mechanism on the feature maps differs accordingly. To determine the optimal insertion point for the attention mechanism, we systematically conducted experiments on the three positions and performed comparative analyses based on multiple indicators (Table 3). The experimental results show that integrating the triplet attention module at different positions can improve detection performance over the baseline YOLOv8s, though the degree of improvement varies. The most substantial performance was observed when the module was incorporated at Position B, resulting in an increase of 0.025 in AP and 0.021 in AR.

**Table 3:** Incorporating the triplet attention module to different positions of YOLOv8s

Position	mAP(0.5)	AP	AR	Macro F1	FPS (f/s)
YOLOv8s	0.945	0.694	0.754	0.884	120
YOLOv8s+TriA_PosA	0.933	0.707	0.767	0.880	113
YOLOv8s+TriA_PosB^※^	0.949	0.719	0.775	0.892	113
YOLOv8s+TriA_PosC	0.942	0.718	0.773	0.892	113
YOLOv8s+TriA_PosBC	0.941	0.710	0.767	0.885	107
YOLOv8s+TriA_PosABC	0.946	0.706	0.761	0.876	101

※: It was selected as the insertion position for the triplet attention module

In fact, though introducing attention modules barely affects model size or computational complexity (GFLOPs), this introduction generates a decline in inference speed. Specifically, the addition of a single attention module reduces the FPS from 120 to 113, and further integration of three modules results in an additional decline to 101 FPS. Based on these findings, we selected B as the optimal position for integrating the triplet attention module, as it provides the most favorable balance between detection accuracy and computational efficiency.

### Effect balance experiment of DSC2f module

As shown in Fig. 8, positions D, E, F and G in the backbone of YOLOv8s are the C2f layers. Given that the backbone network contains the richest feature information, and the introduction of DSC2f will inevitably cause some loss of feature information, which then leads to a negative effect on the model performance. Therefore, besides all C2f modules in the neck were substituted with DSC2f modules, the C2f modules in the backbone were optimized to DSC2f modules one by one to achieve an optimal model architecture (Table 4). The results reveal that substituting C2f modules at Positions D, E with DSC2f modules yields optimal detection performance. This configuration generates significant improvements in key indicators, including mAP(0.5) and macro F1 score, outperforming all other modified variants. Notably, the model maintains computational efficiency with a compact size of 7.76 MB, 22.56 GFLOPs, and an inference speed of 125 FPS. It effectively mitigates potential latency from the attention mechanism while even surpassing the baseline YOLOv8s in speed.

**Table 4:** Replacing C2f modules in different layers of YOLOv8s+TriA_PosB with DSC2f modules

Position	mAP(0.5)	Macro F1	Model size (M)	GFLOPs (G)	FPS (f/s)
DSC2f_Neck	0.944	0.903	9.43	25.26	121
DSC2f_Neck+PosD	0.942	0.902	8.32	24.37	123
DSC2f_Neck+PosDE^※^	0.961	0.926	7.76	22.56	125
DSC2f_Neck+PosDEF	0.933	0.892	7.62	20.80	128
DSC2f_Neck+PosDEFG	0.935	0.896	7.61	19.92	130

※: It was selected as the final strategy for introducing DSC2f modules

Further experiments replaced all the C2f modules in Positions D, E, F and G, resulting in a marginal decrease in model size. Compared to the optimal configuration, it achieved only a 0.15 MB decrease in model size, while the mAP(0.5) and macro F1 scores of this configuration decreased by 0.026 and 0.03, respectively. Consequently, we selected replacing C2f modules with DSC2f in the neck and Positions D, E as the optimal strategy, which balances model size, computational efficiency, and detection accuracy.

### Ablation experiments

**Table 5:** Ablation experiment results

Models	TriA	DSC2f	mAP(0.5)	AP	AR	Macro F1	Model size (M)	GFLOPs (G)	FPS (f/s)
YOLOv8s (Baseline)	○	○	0.945	0.694	0.754	0.884	11.17	28.82	120
YOLOv8s-T	√	○	0.949	0.719	**0.775**	0.892	11.17	28.82	113
YOLOv8s-D	○	√	0.937	0.703	0.759	0.899	**7.76**	**22.58**	**130**
YOLO-RBSD (Proposed)	√	√	**0.961**	**0.721**	0.774	**0.926**	**7.76**	22.59	125

*TriA* Triplet attention module, *DSC2f * Depth-wise Separable C2f module. ○ The module was not added, √ The module was added. Text in bold denotes the top-1 performance among four models for that particular indicator.

To rigorously evaluate the efficacy of the proposed modules, we performed systematic ablation studies (Table 5). The experimental results demonstrate that integrating the triplet attention mechanism substantially improves the detection performance of the YOLOv8s model, with notable enhancements in precision, recall, and the comprehensive macro F1 score. However, this enhancement is accompanied by a slight reduction in detection speed. Given the critical importance of rapid detection in a spore monitoring model, we incorporated the lightweight DSC2f modules based on depthwise separable convolution into YOLOv8s. The results demonstrate that the DSC2f module achieves significant model compression, reducing the model size from 11.17 M to 7.76 M and computational complexity from 28.82G to 22.58G. Moreover, the FPS increases by 10 frames per second compared to YOLOv8s, confirming the dual capability of the DSC2f module in model compression and speed enhancement.

Furthermore, the proposed YOLO-RBSD achieves an optimal balance between detection accuracy and speed. Specifically, its mAP(0.5) value increases by 0.016, AP by 0.027, AR by 0.02, and the macro F1 score by 0.042. These improvements make YOLO-RBSD the top-performing model in this test, even surpassing all original variants of YOLOv8.

### Evaluation of attention mechanisms combined with DSC2f modules

**Table 6:** Performance comparison of four attention mechanisms combined with DSC2f modules

	mAP(0.5)	AP	AR	Macro F1	Model size (M)	GFLOPs (G)
CBAM+DSC2f	0.929	0.709	0.768	0.892	7.77	22.59
SE+DSC2f	0.934	0.715	0.772	0.894	7.77	**22.58**
ECA+DSC2f	0.932	0.708	0.769	0.896	**7.76**	**22.58**
TriA+DSC2f	**0.961**	**0.721**	**0.774**	**0.926**	**7.76**	22.59

In ablation experiments, the introduction of a single module yielded inferior performance compared to the combined integration of both modules. To validate the effectiveness and distinctiveness of this combination, this study compared the performance of three typical attention mechanism, including CBAM [30], SE [31], ECA [32], and the triplet attention, integrated with DSC2f respectively. The results are presented in Table 6. It was observed that the introduction of attention mechanisms had a negligible impact on model size and computational cost. However, substantial differences emerged in detection performance. The high-effective channel attention mechanism ECA underperformed relative to the conventional channel attention mechanism SE, with all evaluation indicators lower than those of SE. In contrast, the two attention mechanisms CBAM and triplet attention that jointly model spatial and channel attention exhibited more pronounced performance disparities when combined with DSC2f. The CBAM + DSC2f combination achieved an mAP(0.5) of 0.929 and a Macro F1 of 0.892, whereas the triplet attention + DSC2f combination attained the best performance, with an mAP(0.5) of 0.961 and a Macro F1 of 0.926, surpassing the former by 3.2% and 3.4%, respectively.

### Comparison with the mainstream algorithms

In this study, a comparative analysis was performed between the proposed method and nine representative object detection algorithms. Among these algorithms, there are two two-stage detection algorithms, including Faster RCNN [10] and Cascade RCNN [11], and five one-stage detection algorithms, including SSD [12], RetinaNet [13], YOLOv9 [33], YOLOv10 [34], YOLOv11 [28], and YOLOv12 [35]. In addition, the Real Time Detection Transformer (RT-DETR) [36] was also selected. As shown in Table 7, a comprehensive evaluation of ten algorithms was conducted, including the evaluation of model accuracy, parameters and speed.

The results show that the previously proposed single-stage algorithms SSD and RetinaNet perform inadequately. The SSD obtains an AP of 0.354 and a macro F1 score of 0.368, ranking it last among all algorithms. The mAP(0.5) of RetinaNet is merely 0.616, also the lowest in comparison to other algorithms. Additionally, the performance of RT-DETR is rather mediocre, as it falls short of the YOLO series in both detection accuracy and speed. Comparing to the above algorithms, two-stage methods such as Faster RCNN and Cascade RCNN excel in detection accuracy. However, their framework, which first generates candidate boxes and then performs classification and localization, limits their detection speed and causes significant computational redundancy. This study aims to develop a model that balances both accuracy and speed. Apparently, two-stage algorithms cannot meet this requirement.

**Table 7:** Comparison of YOLO-RBSD with the mainstream object detection algorithms

Models	Backbone	Map (0.5)	AP	Macro F1	Params (M)	GFLOPs (G)	FPS (f/s)
Faster RCNN	ResNet50	0.914	0.675	0.877	41.36	69.01	63
Cascade RCNN	ResNet50	0.917	0.689	0.882	69.16	96.07	47
RT-DETR	ResNet50	0.82	0.595	0.787	40	125.60	46
SSD	VGG16	0.677	0.354	0.368	26.29	62.75	101
RetinaNet	ResNet50	0.616	0.417	0.528	37.97	170.01	44
YOLOv9s	CSPDarkNet^a^	0.902	0.686	0.874	**6.84**	26.7	70
YOLOv10s	CSPDarkNet	0.910	0.691	0.865	7.66	24.5	98
YOLOv11s	CSPDarkNet	0.929	**0.721**	0.92	8.98	21.3	109
YOLOv12s	CSPDarkNet	0.900	0.687	0.865	8.81	**21.2**	95
YOLO-RBSD	CSPDarkNet	**0.961** ^b^	**0.721**	**0.926**	7.76	22.56	**125**

^a^Although each selected version of YOLO has made improvements to the backbone network, its core architecture has not changed and is still based on Cross Stage Partial Darknet (CSPDarkNet)

^b^Text in bold denotesthe optimal results for that evaluation indicator

In contrast, the YOLO series demonstrates commendable performance in both speed and accuracy. Notably, both YOLOv11s and the proposed YOLO-RBSD exhibited even more significant performance. Specifically, YOLO-RBSD achieved the highest scores in AP, mAP(0.5), and F1 score. Its mAP(0.5) reached 0.961, representing a 3.2% improvement over YOLOv11s. The superior performance of YOLO-RBSD further validated the effectiveness of the combination of the triple attention modules and DSC2f modules. Although the model size of YOLO-RBSD is not the smallest within the YOLO series, it is only 0.92 M larger than the smallest variant YOLOv9s (6.84 M). Meanwhile, its computational load is lower than that of YOLOv9s, and it can process 125 images per second, making it the fastest among all algorithms. In conclusion, YOLO-RBSD exhibits the best overall performance among all comparative algorithms.

### Visualization of detection results

**Fig. 9:**
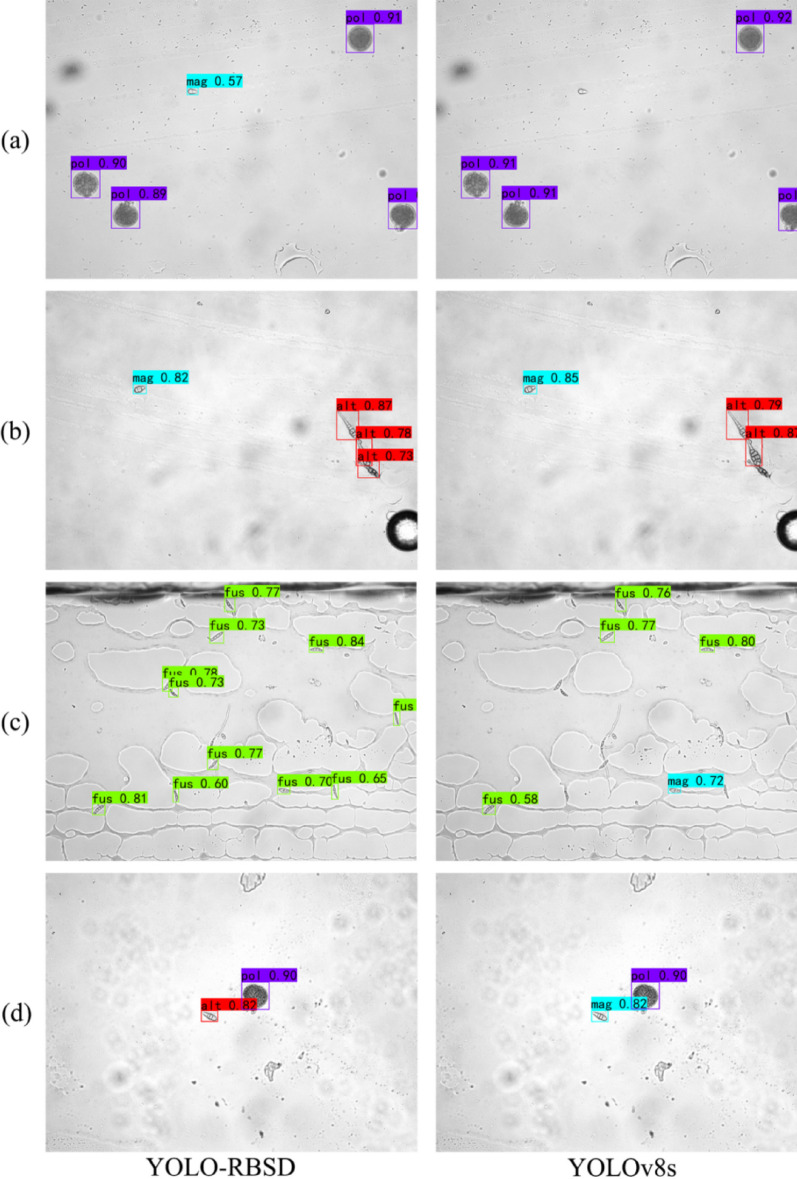
The examples of model detection outputs generated by YOLO-RBSD and the baseline model YOLOv8s

**Fig. 10:**
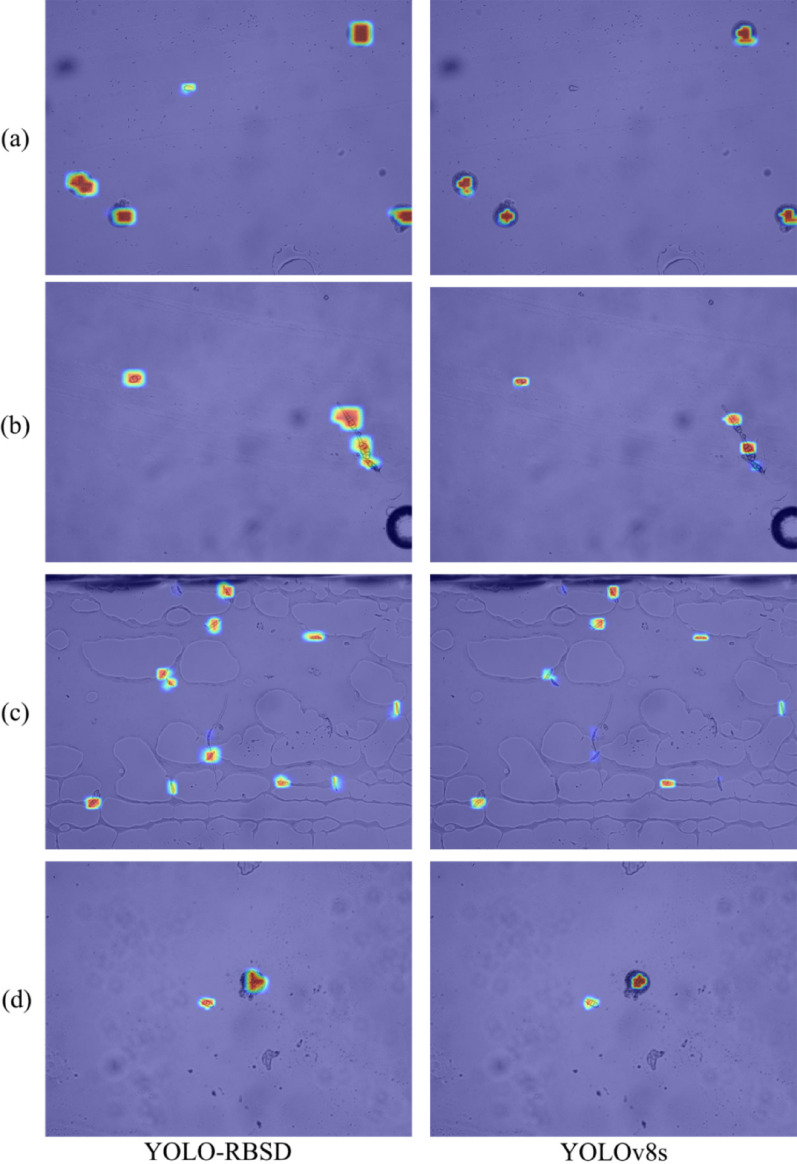
The samples of heatmap generated by YOLO-RBSD and the baseline model YOLOv8s. The confidence scores for specific areas in the image will be displayed in order from high to low as red, yellow, green, blue, and purple

To further evaluate the performance enhancement of the proposed model in spore detection, detection outputs (Fig. 9) and corresponding heatmaps (Fig. 10) were generated for YOLO-RBSD and the baseline model YOLOv8s on the test set, with four representative samples selected for visualization. The output samples indicate that the baseline model YOLOv8s frequently experiences missed detections and false detections, while YOLO-RBSD can accurately identify spore types, even in complex image backgrounds or overlapping spores (Fig. 9).

When dealing with small objects (Fig. 9a and c) or overlapping instances (Fig. 9b), YOLOv8s tends to miss detections. The corresponding heatmaps (Fig. 10a, b and c) show that the undetected object areas appear as purple or blue regions with low confidence score. Additionally, when spores share certain morphological similarities, YOLOv8s frequently misclassify them into incorrect categories (Fig. 9c and d). For instance, in Fig. 9d, YOLOv8s mistakenly identified a non-standard alt spore as a rice blast spore (mag). Although the two spores are morphologically similar, rice blast spores are typically more fusiform and have only 1–2 septa, whereas the alt spore exhibits 3 septa and an sunflower-seed-like shape. Instead, YOLO-RBSD is able to capture the subtle distinctions between them, enabling correct classification. Furthermore, the heatmaps also reveal that YOLOv8s mostly captured partial features of the objects, resulting in irregular activated regions (Fig. 10). In contrast, YOLO-RBSD produced larger red or yellow pixel areas (indicating high activation) that fully covered each object, highlighting its precise capture of high-dimensional features.

### Comparison of detection results on four categories

**Fig. 11:**
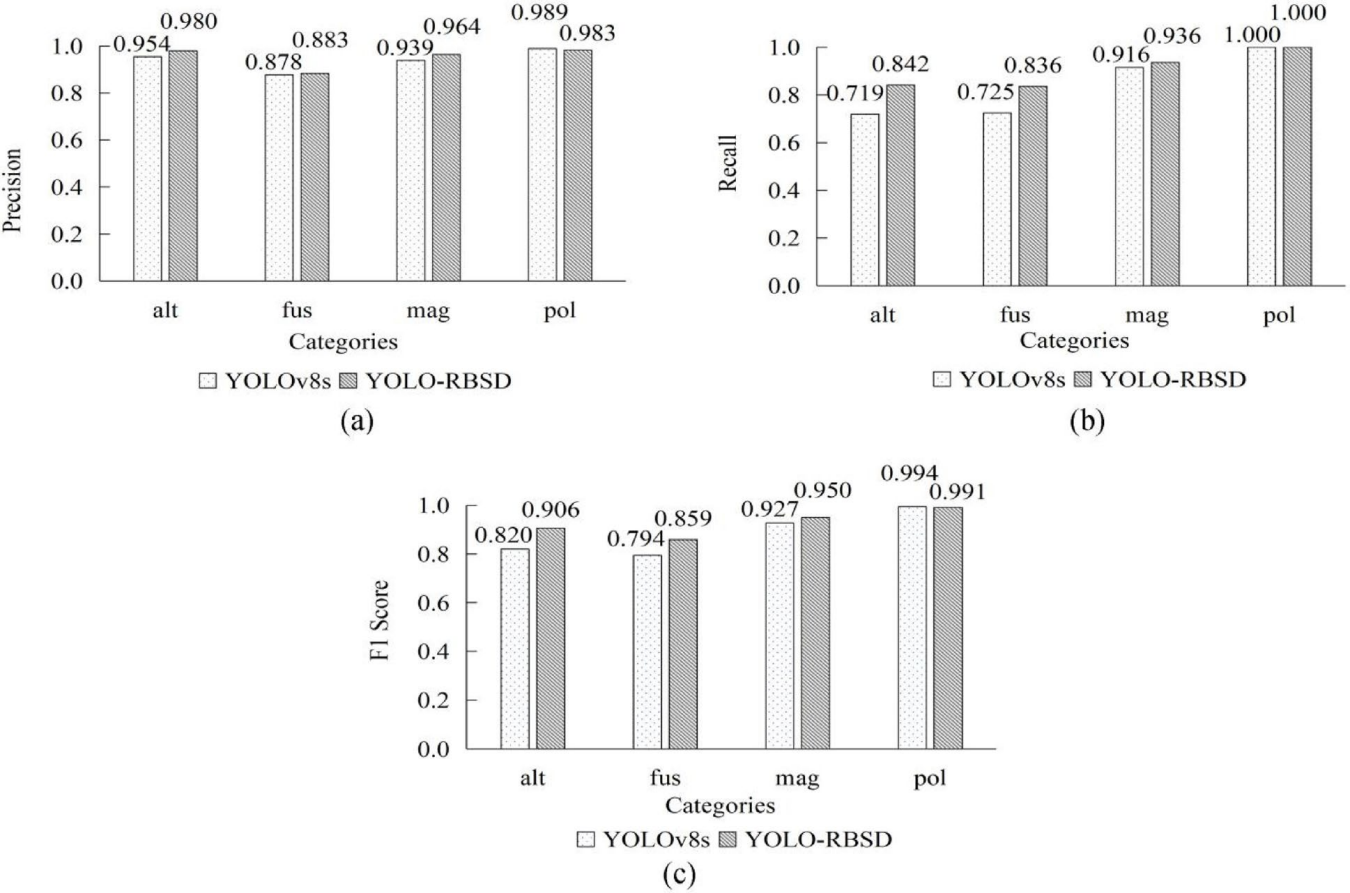
Performance comparison between YOLO-RBSD and baseline model YOLOv8s on four categories of the spore dataset. **a** Precision, **b** Recall, **c** F1 score. *alt Alternaria* spp., *fus Fusarium* spp., *mag Magnaporthe oryzae*, *pol* rice pollon

**Fig. 12:**
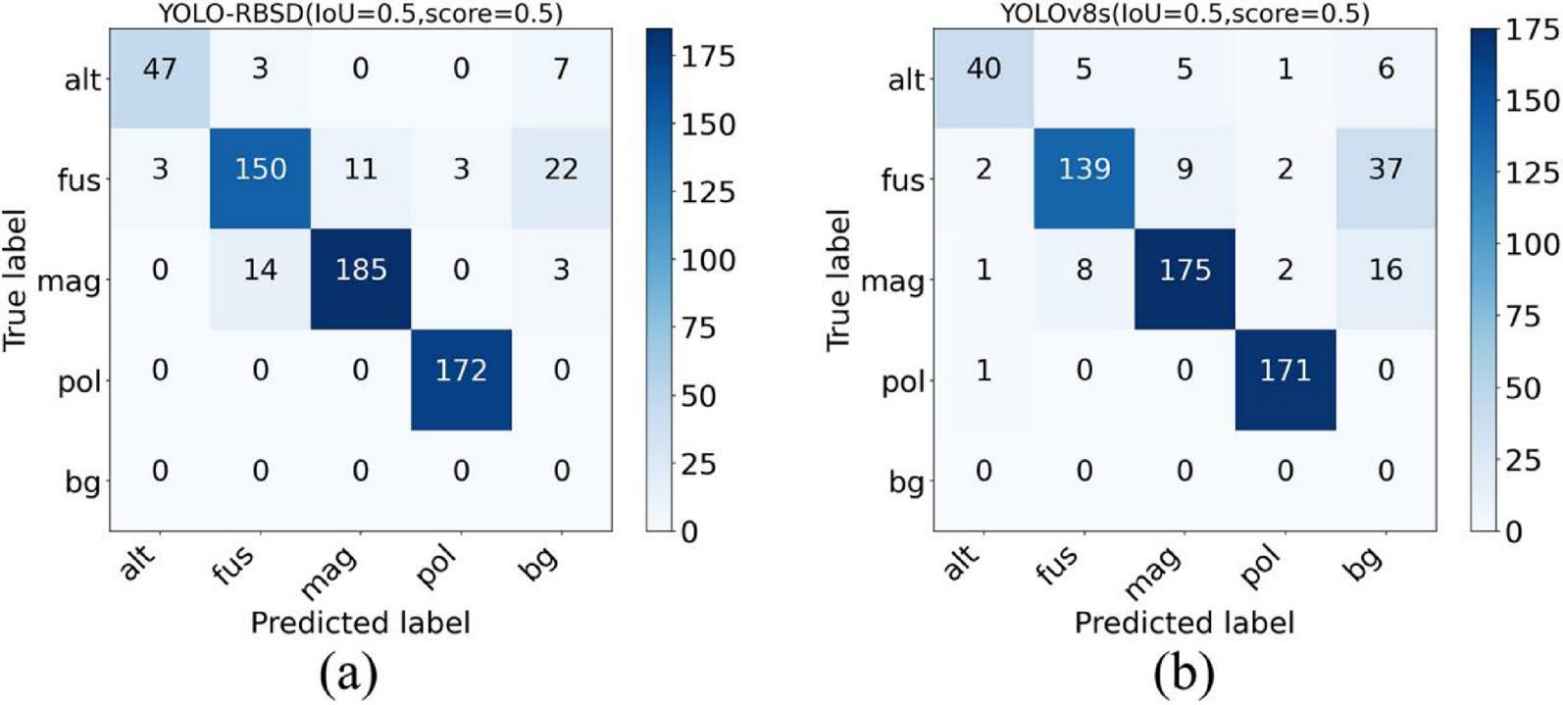
Confusion matrices of the proposed YOLO-RBSD and the baseline YOLOv8s on the test set. *alt Alternaria* spp., *fus Fusarium* spp., *mag Magnaporthe oryzae*, *pol* rice pollon, *bg* background

To demonstrate the model detection performance across different categories, we calculated and statistically analyzed the precision, recall, and F1 score of YOLO-RBSD for the four categories. These results were then compared with the corresponding indicators of YOLOv8s (Fig. 11). Among the four categories, the model performed relatively poorly on alt and fus. To further investigate the causes of the relatively poor detection performance for the two categories, confusion matrices of two models based on the test set results were constructed (Fig. 12). The confusion matrices reveal that the instance count for alt is the lowest among all categories, a finding consistent with the statistical results presented in Table 1. Moreover, alt exhibits substantial morphological variation across different developmental stages. The limited number of instances hinders comprehensive feature learning for this category, thereby reducing detection accuracy. The other underperforming category was fus, its smaller size and crescent shape make it easily confused with mycelium or plant tissue, leading to frequent misidentification as background and consequently a low recall rate.

The model achieved higher detection rates for mag and pollen, with pollen attaining a particularly remarkable recall of 100%. This study primarily focused on detecting rice blast spores, and Fig. 11 indicates that both models achieved high precision and recall on this category, as well as high F1 score. Nevertheless, it shows that YOLO-RBSD performed better on the four categories, outpacing YOLOv8s on all three indicators. Consistent findings for both models are illustrated in the confusion matrices (Fig. 12).

## Discussion and future work

For object detection algorithms, the construction and preprocessing of datasets often significantly impact model performance. To enhance sample diversity and improve model generalization, this study collected samples from three cities across different years and utilized more than two types of microscopic imaging devices for image acquisition. Meantime, the dataset is a real spore microscopic images captured in field environments, characterized by scattered targets, shriveled morphologies, and numerous impurities. Models trained on this dataset demonstrate effective performance on samples collected under complex field conditions, indicating high practical value. However, the current dataset still requires further expansion. For instance, rice blast spores from different regions exhibit subtle morphological variations, and impurities collected during the same period may vary depending on the rice disease stage. To further enhance the generalization ability of models, we will continue to collect samples from other rice-producing regions for training and testing. Additionally, during data annotation, a small number of hard samples may exists, these objects are challenging for the model to correctly identify [37]. Improving the detection capability of models for these samples can further enhance its overall performance. Therefore, in the future, we plan to optimize the proposed model specifically for hard samples or employ knowledge distillation techniques to strengthen the model ability.

Among the different configurations of triplet attention module, inserting it into the connection point between the backbone P4 layer and the neck yielded relatively superior detection performance. This may be attributed to the fact that P4, as a deeper layer, carries richer three-dimensional feature representations than P3. Additionally, since the model upsamples the convolutional layers after applying attention module, the enhanced features from P4 can positively affect the feature fusion of P5. Many studies, when modifying YOLO-series models, tend to import enhancement modules directly to the exit of the last layer of the backbone network, while neglecting the interaction intensity of feature information across different dimensions [38, 39]. However, this study demonstrates that the addition position of enhancement modules also exerts a significant impact on the overall performance of the model, providing a new approach for the effect evaluation of subsequent model improvements.

The introduction of the lightweight DSC2f module effectively reduces the number of model parameters, and facilitates the deployment of the model on various devices, especially on mobile devices. Many studies on improved YOLO algorithms achieve either a reduction in model parameters or an increase in detection speed, but rarely both [17, 18]. However, the DSC2f module is capable of lowering the model parameters while also enhancing detection speed by lowering computational complexity. Despite showing only a modest speed gain over the baseline and YOLOv11s in testing, this improvement saves considerable time on larger datasets, facilitating rapid assessment of field spore density. Moreover, the introduction of the DSC2f module not only maintained model performance in spore detection but also enhanced detection effectiveness when combined with the attention mechanism.

A key weakness lies in the existing attention mechanisms, such as channel-wise SE and ECA, which primarily focus on modeling channel dependencies while neglecting cross-dimensional spatial-channel interactions. This limitation severely restricts their ability to capture the anisotropic morphologies of spores, such as elliptical, filamentous, or irregular shapes, which require simultaneous modeling of height, width, and channel relationships. Although CBAM incorporates both spatial and channel attention, it lacks interaction across different dimensions. The triplet attention mechanism emphasizes inter-dimensional interaction, thereby providing richer informational exchange during the feature fusion process within the neck network. Furthermore, although depthwise separable convolution reduces computational complexity to some extent, it inevitably loses certain feature information. The triplet attention mechanism compensates for this loss by enhancing interaction across dimensional information through the rotation of three-dimensional features, ultimately achieving superior feature extraction performance.

After comparison, we found that the current trend in the YOLO series leans towards integrating attention modules to enhance detection performance, such as the C2PSA module in YOLOv11 and the area attention mechanism in YOLOv12. Their successive breakthroughs on generic datasets further underscore that attention mechanisms have gradually become a crucial strategy for boosting model performance. However, in practical applications within specialized fields such as industry, medicine, or agriculture, there are often unique data formats and specific object categories [40–42]. Therefore, it is important to select the most suitable attention mechanism for these specific tasks. In addition, we also found that importing NMS-free strategy or lightweight modules can effectively improve the model speed. For instance, YOLOv10 was the first to import the NMS-free strategy in YOLO series, obtaining a significant increase in detection speed compared to the earlier versions. And YOLOv11 incorporated the lightweight C3K2 modules to reduce computational load. In this study, YOLO-RBSD has a unique dual-module design, and this design can improve both accuracy and speed at the same time.

Notably, we primarily focused on optimizing the backbone and neck networks in this study, while overlooking optimization in the prediction head. The NMS strategy used for outputting prediction boxes significantly reduces the processing speed. The latest version of the Ultralytics series, YOLOv26 [43], has already removed this algorithm, and the DETR series also does not rely on NMS, thereby effectively enhancing their inference speed. It would be an approach worthy of future reference. Meanwhile, one of the latest algorithms in the DETR series, RF-DETR [44], is currently being evaluated across various tasks. Its Weight-Sharing Neural Architecture Search (Weight-Sharing NAS) strategy allows for network structure adjustments tailored to different application objectives, which may reshape future research workflows. The application of spore detection models would no longer be constrained by hardware limitations and could be deployed more efficiently to edge devices.

According to the plant disease triangle theory, host plants, the environment and pathogens constitute the three essential elements for the occurrence of rice blast disease [45]. Previous rice blast monitoring systems primarily relied on meteorological data, failing to incorporate spore quantity for a more accurate assessment of disease occurrence and development [46]. The limitation arises from the lack of feasible methods for measuring the airborne spore concentration of rice blast in the field. This study not only proposes a novel spore detection algorithm but also establishes a method for measuring the amount of spores floating in the air, which includes in-field spore trapping, data collection, dataset construction, and spore detection. The specified equipment and experimental details employed in this method have undergone extensive testing and validation. The successful implementation of this method offers valuable insights for the development of subsequent monitoring systems for rice blast spore density.

## Conclusions

In this study, we developed a microscopic image dataset comprising rice blast spores and three types of impurity particles, and proposed a deep learning-based rice blast spore detector named YOLO-RBSD. This detector integrates a triplet attention module into the framework and replaces partial C2f modules with parameter-efficient DSC2f modules. The modified model exhibits significant improvements in both accuracy and speed, achieving a mAP(0.5) of 0.961, a macro F1 score of 0.926, and an image processing speed of 125 FPS, which collectively surpass the baseline and mainstream models. Furthermore, the model visualization analysis demonstrates that YOLO-RBSD can capture high-dimensional features of spores and reduce the miss rate and false detection rate. This study addresses the automatic spore counting issue, which is the key technical challenge of the low-altitude rice blast spore density monitoring system. However, the monitoring system needs further improvements for real application. In the future, we will explore the correlation between pathogen amount and rice blast incidence. The establishment of this monitoring system can provide farmers with timely pathogen amount data, enabling farmers to apply pesticides for prevention and control promptly. It serves as a powerful tool for short-term forecasting of rice blast and offers data support and historical reference for understanding the epidemic patterns of rice blast in affected regions.

## Supplementary Information

Supplementary Material 1.

## Data Availability

The datasets used and analyzed during the current study are available from the corresponding author on reasonable request.
