# Supplementary material for: YOLO-RBSD: an efficient and accurate rice blast spore detector based on improved YOLOv8
Source: Plant Methods. 2026 Apr 5;22:34. doi: 10.1186/s13007-026-01526-5 (PMC13059388; doi:10.1186/s13007-026-01526-5)
Supplement: Supplementary file 1 — Supplementary Material 1. [file 13007_2026_1526_MOESM1_ESM.docx]

**Supplementary materials**


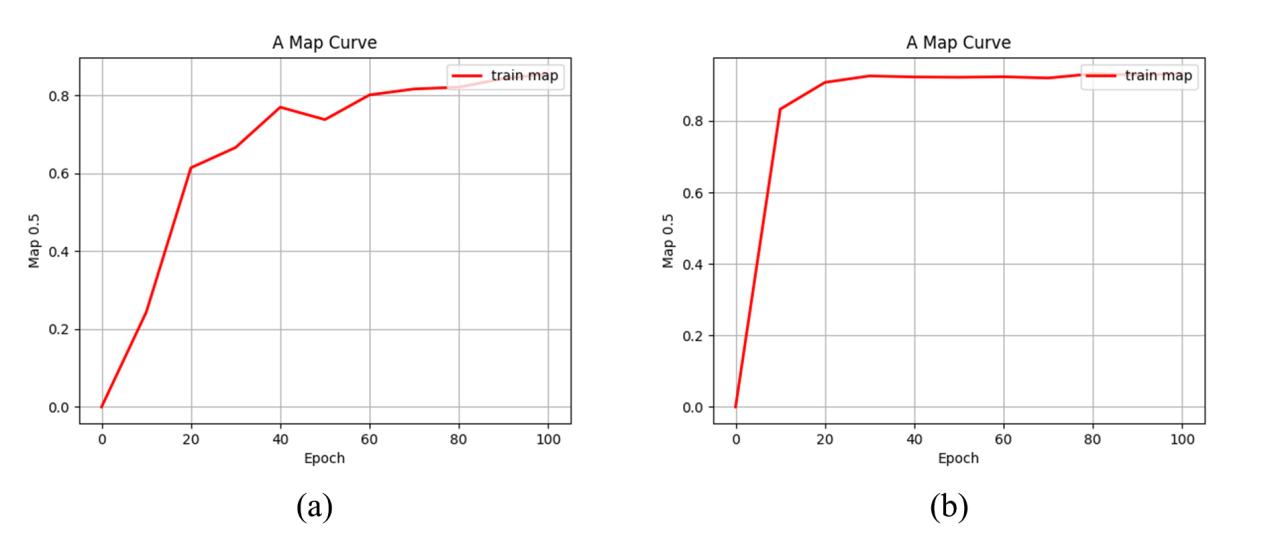


**Fig. S1** The progression of mAP(0.5) for YOLO-RBSD models with and without COCO dataset pre-training over 100 training epochs. (a) YOLO-RBSD without pretraining, (b) YOLO-RBSD with pretraining.
